# The Optimization of Dispersion and Application Techniques for Nanocarbon-Doped Mixed Matrix Gas Separation Membranes

**DOI:** 10.3390/membranes12010087

**Published:** 2022-01-13

**Authors:** Ruben Hammerstein, Tim Schubert, Gerd Braun, Tobias Wolf, Stéphan Barbe, Antje Quade, Rüdiger Foest, Dionysios S. Karousos, Evangelos P. Favvas

**Affiliations:** 1 Institute of Chemical Process Engineering and Plant Design, TH Köln, 50679 Cologne, Germany; ruben.hammerstein@th-koeln.de (R.H.); gerd.braun@th-koeln.de (G.B.); 2 Faculty of Applied Natural Sciences, Chemical Engineering, TH Köln, 51368 Leverkusen, Germany; tobias.wolf1@th-koeln.de (T.W.); stephan.barbe@th-koeln.de (S.B.); 3 Leibniz-Institute for Plasma Science and Technology e.V. (INP), 17489 Greifswald, Germany; quade@inp-greifswald.de (A.Q.); foest@inp-greifswald.de (R.F.); 4 Institute of Nanoscience and Nanotechnology, National Center for Scientific Research “Demokritos”, Aghia Paraskevi, 15341 Athens, Greece; d.karousos@inn.demokritos.gr (D.S.K.); e.favvas@inn.demokritos.gr (E.P.F.)

**Keywords:** mixed matrix membranes (MMMs), supported thin films, cellulose acetate, polyimide, CNT dispersion, plasma treatment, three-roll-mill (TRM), rotor-stator system (RS), spin coating, gas separation

## Abstract

In this work, supported cellulose acetate (CA) mixed matrix membranes (MMMs) were prepared and studied concerning their gas separation behaviors. The dispersion of carbon nanotube fillers were studied as a factor of polymer and filler concentrations using the mixing methods of the rotor–stator system (RS) and the three-roll-mill system (TRM). Compared to the dispersion quality achieved by RS, samples prepared using the TRM seem to have slightly bigger, but fewer and more homogenously distributed, agglomerates. The green γ-butyrolactone (GBL) was chosen as a polyimide (PI) polymer-solvent, whereas diacetone alcohol (DAA) was used for preparing the CA solutions. The coating of the thin CA separation layer was applied using a spin coater. For coating on the PP carriers, a short parameter study was conducted regarding the plasma treatment to affect the wettability, the coating speed, and the volume of dispersion that was applied to the carrier. As predicted by the parameter study, the amount of dispersion that remained on the carriers decreased with an increasing rotational speed during the spin coating process. The dry separation layer thickness was varied between about 1.4 and 4.7 μm. Electrically conductive additives in a non-conductive matrix showed a steeply increasing electrical conductivity after passing the so-called percolation threshold. This was used to evaluate the agglomeration behavior in suspension and in the applied layer. Gas permeation tests were performed using a constant volume apparatus at feed pressures of 5, 10, and 15 bar. The highest calculated CO_2_/N_2_ selectivity (ideal), 21, was achieved for the CA membrane and corresponded to a CO_2_ permeability of 49.6 Barrer.

## 1. Introduction

Membrane-based gas separation is an interesting technology for a multitude of tasks regarding CO_2_ separation for natural gas sweetening [1], biogas treatment, and carbon capturing [2]. The CO_2_ separation for carbon-capturing might mostly be applied in the industrial combustion processes, either as a pretreatment of the fuel (e.g., CO_2_/CH_4_) or as a waste gas treatment (CO_2_/N_2_) [2]. The background of this study was on flue gas treatment, and, therefore, the separation of CO_2_ from N_2_ in a vacuum-driven membrane configuration was investigated.

The use of membrane processes has increased due to their benefits of low energy and production costs, ecofriendliness, and a simple scale-up because of the module-based operation units [3,4]. To require the different separation requirements, two main membrane types are used, known as polymeric and inorganic membranes. Polymeric membranes are widely applied in industrial gas separation due to their beneficial properties, such as good mechanical resistance and high availability [5]. However, undesirable effects such as the trade-off limit between permeability and selectivity [6] and plasticization effects caused by acid gases hinder the widespread utilization of polymeric membranes in CO_2_ separation [1,4]. Inorganic membranes, on the other hand, exhibit excellent gas separation properties well above the trade-off limit of polymeric membranes and are stable at high temperatures, but they are not manufacturable in sufficient quantities/sizes for an actual reasonable industrial application [7]. The mixed-matrix membranes (MMMs) are an approach that combines the gas separation properties of inorganic particles, e.g., molecular sieve particles, with the advantageous properties of a polymeric matrix [8]. Different studies regarding a multitude of possible filler materials in different polymers were conducted and the optimized integration of adding the fillers into the matrix to enhance the gas separation properties of MMM is still a challenge. Besides polysulfone (PSF) [9] and polpolyethylene glycol (PEG)/polyethersulfone (PES) blends [10,11], promising polymeric matrixes for the separation of CO_2_ are cellulose acetate (CA) and polyimide (PI), because of the high solubility of CO_2_ in CA and the high separation performances of PI [1,3,12,13]. With regard to circularity, CA has the benefit of being considered a green polymer with high availability [13].

Utilizing the spin coating process, thin layers can be created by applying a liquid solution onto a substrate that is then rotated with high acceleration. The solution is discharged from the substrate by centrifugal forces until an equilibrium with the viscous resistance of the fluid is reached and a thin film is formed [14]. After reaching this equilibrium, the film thickness further shrinks, due to the evaporation of the solvent, until the film dries into a solid layer [15]. Spin-coating was first performed with a model in 1958 by Emslie et al. [16] that described the coating using a Newtonian fluid. Numerous other models have been described for non-Newtonian liquids and specific applications [14,17,18,19]. Today, spin-coating is broadly applied in semiconductor manufacturing for the deposition of photoresists on silicon wafers; however, other applications that demand the formation of thin films, such as membrane preparation, have only rarely been described in the literature before [20]. Therefore, a fairly new method of membrane preparation is described in this work.

## 2. Materials and Methods

**Figure 1:**
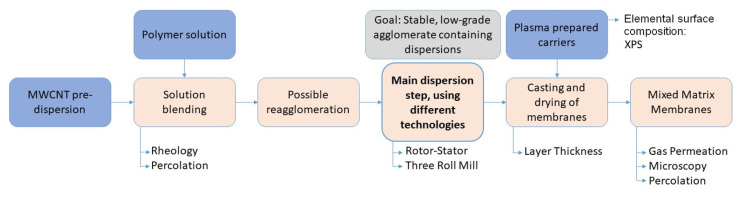
Schematic representation of the intermediate steps for mixed matrix membrane production and characterization methods.

For the preparation of asymmetric mixed matrix membranes, a dispersion of carbon nanotubes (CNTs) in polymeric solution was coated on carrier substrates using a spin coating process. Dispersions were made by the solution blending method with an additional dispersion step to avoid agglomeration. The status of the dispersion quality was evaluated by electric percolation considerations and microscopic analyses. After coating, the membranes were dried and the permeation properties were analyzed for CO_2_ and N_2_. The membrane preparation process is illustrated in Figure 1.

### 2.1. Solution Blending

The first step of the membrane preparation process was the solution blending. A polymeric solution was mixed with additives that were predispersed in the same solvent. The polymeric solution was made by dissolving cellulose acetate (CA) powder (Daicel, Japan) in diacetone alcohol (DAA) (Alfa Aesar, Tewskbury, MA, USA) under vigorous stirring with a horseshoe or paddle agitator. Multi-walled carbon nanotubes (MWCNT: FutureCarbon, Bayreuth, Germany) were dispersed into the DAA using a high-pressure homogenizer. The solution and the predispersed MWCNTs were slowly mixed and stirred until a homogenous solution was obtained. The preparation of the CA solution and the solution blending both were done at room temperature. Solution blending is a critical step that leads to reagglomeration in many cases. 

Additional experiments were conducted with the alternative polymer system, Polyimide (PI) P84 (Ensinger, Lenzing, Austria). PI is not soluble in DAA; therefore, γ-Butyrolactone (GBL) (Merck, Darmstadt, Germany) was used as the solvent. Additional MWCNT predispersions (FutureCarbon) based on GBL were used for solution blending. The preparations of solutions and solution blending were both done in a similar way to the methods used for CA.

During solution blending, an additional solvent was introduced together with the CNTs, lowering the concentration of polymers in the final dispersions. All predispersions had a CNT concentration of 1.5 wt.%. The amount of predispersion mixed with the solution was determined by the polymer concentration, to achieve a CNT concentration of 2 wt.% regarding the dry mass (the final membrane). The final composition of the dispersions prepared from the solutions with different polymer concentrations is specified in Table 1. Throughout the course of this work, the dispersion will be identified by the polymer concentration of the solution they were prepared from.

### 2.2. Main Dispersion Step

To avoid the reagglomeration of the MWCNTs, an additional mechanical dispersion was applied after solution blending. For this step, a rotor–stator-system (RS) (Silverstone, GB) and a three-roll-mill (TRM) 80E (Exakt, Norderstedt, Germany) were used. Due to the intense heating of the dispersions, the RS was fitted with a cooler for the beakers, operating at 15 °C. The temperature control unit of the TRM was set to 20 °C. A detailed study of the dispersion process is described in [13].

In the RS, the dispersion is agitated by a stirrer that is encased in a radial-mounted stator with a narrow gap between the two. The high-speed rotation of the rotor induces mixing energy and shear stress at a higher rate compared to conventional agitators [21]. In this study, a L5M-A (Silverson, GB) with a blade design [22] rotor and a radial emulsion sieve style [21] stator was applied. The outer diameter of the rotor was 31.2 mm and the inner diameter of the stator was 31.6 mm, resulting in a gap size of 0.2 mm. One hundred grams of the polymer solution and MWCNT predispersions were manually mixed in a beaker, which was then placed in a cooling bath set to 15 °C. The head was immersed in the mixture and placed in the center of the fluid body, with a distance of at least 1 cm from the bottom of the beaker and the surface of the mixture. After switching on the RS, the speed was slowly increased to 6000 rpm and automatically stopped after 30 min.

The TRM generates a shear force by the differential speed between the rolls that counter-rotate with narrow gaps between them. One important characteristic of the dispersion in the TRM is that it is almost exclusively induced by local shear forces with a short residence time [23]. The TRM is equipped with 80 mm diameter rolls that have a fixed reduction gear ratio of 1:3:9 and a minimum gap width of 5 µm. For the experiments, 100 g of the polymer solution and MWCNT predispersions were manually mixed and slowly poured into the gap between the first and second roll. The mixture accumulated in the space above the gap, where it was further mixed by the rotation of the rolls [24]. In the meantime, a small amount continuously passed through the gap and formed a film on the rolls, which was passed through the gap between the second and third rolls and was then scraped off and collected. This process proceeded until the first gap was empty and was then repeated two more times for every dispersion. The force transmission takes place via the viscous liquid, while the speed gradient in the gap is used to build up the shear force.

The shear rate in both devices can be calculated by Equation (1), where Δ*v* is the characteristic speed gradient in m/s and *δ* is the gap width of the shear gap in m.

(1) $$\dot{\gamma }=\frac{∆v}{\delta }$$

For the RS, the speed gradient is equal to the tip speed of the rotor due to the fixed stator. For the TRM, it is defined by the differential speed of the two neighboring rolls. At the typical operating parameters of 6000 rpm for the RS and 100 rpm (fastest roll) at the 5 µm gap width for the TRM, shear rates of ${\dot{\gamma }}_{RS}=4.9\cdot 10^{4}\;s^{-1}$ and ${\dot{\gamma }}_{TRM}=5.6\cdot 10^{4}\;s^{-1}$ could be achieved. These values fit well within the ranges given in the literature [21,25]. With both dispersion methods being in the same order of magnitude regarding the shear rates, the main difference is the duration of exposure and additional effects, such as possible “cutting” at stator tilts. As mentioned before, the dispersion was mixed and sheared in the RS for 30 min continuously. In the TRM, it was exposed to the shear force only for a short time while passing through the gaps between the rolls.

### 2.3. Substrate (Carrier) Preparation

In contrast to the pretests to establish the spin coating method which was carried out with dense PP substrates, for the membrane preparation, porous carrier membranes were chosen. They provide no significant resistance to permeation and the coated layer determines the permeability and selectivity. The selection of carrier membranes is described in “3.4 Membrane Preparation”. 

The results due to partially not satisfying wetting behavior and adhesion during pretests and membrane preparation steps on the carrier membranes, e.g., separated layers and holes (Section 3.2), evinced the need for a plasma treatment process. 

### 2.4. Plasma Treatment

Plasma-assisted surface functionalization was performed on the membrane carrier material to adjust its wettability. The plasma treatments were performed in a V55G plasma reactor (PLASMA-finish, Wertheim, Germany) consisting of an aluminum vacuum chamber with the dimensions of (40 × 45 × 34) cm^3^ (width × depth × height). A low-pressure microwave discharge operating at a frequency of 2.45 GHz was generated. Details of this plasma device have been published earlier [26]. 

Binary gas mixtures of argon (Ar, 5 sccm) with oxygen (O_2_, 100 sccm) were used as process gases for the plasma functionalization step. The high-purity gases (Ar grade 5.0 and O_2_ grade 4.8) were purchased from Air Liquide.

The microwave power was set to 1200 W for the power input. The pulse-on time of the microwave was fixed at 20 ms with a pulse-off time of 180 ms. The operating pressure was kept constant at 20 Pa. 

The samples were placed 11 cm below the microwave window and were treated for 100 s for effectiveness.

### 2.5. X-ray Photoelectron Spectroscopy (XPS)

The elemental surface composition and chemical binding properties of the plasma-treated samples were analyzed by X-ray photoelectron spectroscopy (XPS) using an AXIS Supra DLD electron spectrometer (Kratos Analytical, Manchester, UK). The spectra were recorded utilizing monochromatic X-rays, Al kα (15 kV, 10 mA for general spectra and 15 kV, 15 mA for highly resolved measured C 1s peaks), with a medium magnification (field of view 2) lens by selecting the slot mode. At a pass, the energy of 160 eV survey spectra were acquired. The a pass energy of 80 eV was used for estimating the chemical elemental composition and 10 eV for the highly resolved measured C 1s peaks for the investigation of the chemical functional groups. Charge neutralization was applied for all samples to reduce any potential differential charging effects. 

Data acquisition and processing were carried out using CasaXPS software, version 2.15 (Casa Software Ltd., Teignmouth, UK). After the subtraction of a Shirley background, the peaks were quantified using a Gaussian–Lorentzian GL (30) peak shape. Highly resolved measured C 1s peaks were fitted using the following components: binding energy (BE) at 285.0 eV: C-C/C-H, BE at 286.55 eV: hydroxyls, ethers (-C-OH/R), BE at 287.8 eV: aldehydes, ketones (-C=O), and BE at 289.4 eV: ester and carboxyl groups (-C-O-C=O, -COOH) [27]. For quantification, an average was calculated from data measured in three different sample spots.

### 2.6. Membrane Preparation

Membranes were prepared by coating a thin layer of dispersion on carriers using a spin coater (SPS, Netherlands) and its subsequent drying. To avoid damages to the equipment caused by GBL, the interior of the spin coater was lined with aluminium foil. First, wetting and adhesion experiments on the coating process were done on “dense” polypropylene (PP) carriers for the CA system and on microscope slides for the PI system. For the actual membranes, a highly PES membrane was used as a carrier in case of the CA system. However, PES is not resistant against GBL; therefore, for the PI system, a porous PP membrane was used. To fit in the spin coater, the carriers were cut into circles with a diameter of 10 cm. In the spin coater, the carriers were fixed to a rotating chuck by vacuum and various volumes of dispersion were applied to them with a syringe that was placed above the center. During the application of the dispersion, the carrier was rotated at a low speed that was increased to the coating speed immediately after. The coating speed was varied between experiments in a range of 2000–8000 rpm. For all experiments, the speed-up from the application speed to the coating speed was done in one second and the coating time was for one minute.

After coating, the wet films were dried at room temperature for 24 h. To prevent the membranes from rolling up due to the tension introduced by the drying film, they were fixated on a flat stainless steel sheet using holding rings. All carriers/membranes were weighed on an analytical balance (Sartorius, Göttingen, Germany) before coating, after coating, and after drying.

### 2.7. The Analysis of Membrane Properties/Permeation Performance

The flow characteristics of the dispersion were analyzed with a rheometer Kinexus pro (Netzsch, Selb, Germany) and an accelerated stability analysis was done in [13]. The dispersion quality of the dried films was evaluated using optical microscopy. For the samples on the PP carriers, a transmitted light microscope BA310E with a Moticam 5+ (Motic, Spain) was used and, due to the PES carriers being too opaque, a reflected light microscope VH-Z100UR (Keyence, Osaka, Japan) was also applied. The pictures were interpreted with the open-source software, ImageJ Fiji [28], applying a black–white balance method, there the agglomerates were detected as dark pixels. This method is limited by the microscope’s resolution of about 1 µm, so only the bigger agglomerates could be observed.

**Figure 2:**
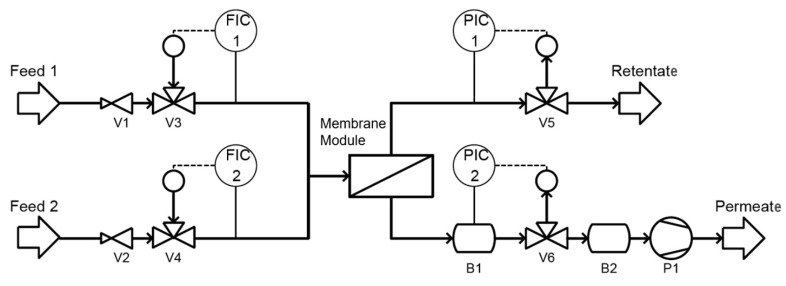
Flow chart of membrane testing rig.

Gas permeation tests were done using a constant volume apparatus [29], illustrated in Figure 2. Here, the permeation was determined by the rate of the pressure increase over time in a vacuum cavity (B1) that was closed off by the membrane, while the other side of the membrane was overflown by the pressurized test gas [29]. Pressure on the vacuum side was measured via the pressure transmitter (PIC2), TST 10 (Tival, Berlin, Germany). The initial pressure on the permeate side was 260 mbar that increased due to permeation. After the pressure in B1 increased by 10 mbar, the solenoid valve (V6) was opened and the pressure was decreased back to the initial pressure, starting the next experiment. The equalizing reservoir (B2) was constantly kept at low pressure to accelerate the pressure decrease. Single gas experiments were conducted with N_2_ and CO_2_ at 5, 10, and 15 bar feed pressures and a flow rate of 200 (norm) L/h. The feed rate was controlled by the flow regulators FIC1/V3 (N_2_) and FIC2/V4 (CO_2_), which were both el-flow F-201CV (Bronkhorst, Netherlands). Pressure on the feed side was regulated by the pressure-reducing valves V1 and V2 and by the pressure regulator, PIC1/V5 el-press P-702CV (Bronkhorst, The Netherlands). 

The membrane module used in this setup was constructed to accommodate the round membranes prepared by spin coating. To enable high-pressure tests, the membrane was placed on a porous sintered metal support that prevented the deformation by the transmembrane pressure differential. The module was sealed by an O-ring on the feed side of the membrane. Therefore, the active area of the membrane was reduced to 42 cm^2^. 

### 2.8. Percolation Study

As described in Section 3.1, the electrical resistivity measurements were part of the characterization of both the suspensions and the applied layers of the membrane, as part of a percolation study to achieve information about filler arrangements and dispersion degrees, as well as the rearrangement of fillers in the matrix during the application and drying process. 

The electrical conductivity of the dispersions was measured by a plate-plate configuration conductivity meter made from two copper electrodes fixed parallel to each other at a defined distance. During the experiments, the dispersion was agitated with a paddle mixer and the probe was submerged in the dispersion. The electrical resistance between the electrodes was measured with a multimeter DM3058E (Rigol, Suzhou, China) and the electrical conductivity was calculated using Equation (2).

(2) $$\sigma =\frac{1}{R}\cdot \frac{l_{E}}{A_{E}}$$

Here, *σ* is the electrical conductivity in S/m, *R* is the resistance in Ohm, *l_E_* is the distance of the electrodes of 2 cm, and *A_E_* is the effective area of the electrodes at 4 cm^2^. During the measurements, the dispersion temperature was regulated to 20 °C using the cooling bath from the RS experiments. To avoid skewing the results by potentially isolating impurities on the electrodes, their surfaces were cleaned before and after every measurement using mild electrolytic pickling.

For the analysis of the solid layers, a probe with four electrodes that were arranged in a line, with defined distances between them, was utilized. Copper needles were used as electrodes. Between the outer two electrodes, a constant direct current was applied, while the potential drop was measured between the inner electrodes [30]. In addition to the multimeter, the power supply, PS 1302D (Voltcraft, Berlin, Germany), was used. The electrical resistance of the layers was calculated from the current I, and the potential drop Δ*U*, by Equation (3) for the application on sheet materials [31].

(3) $$R_{s}=\frac{\pi }{\ln\left(2\right)}\cdot \frac{∆U}{I}$$

From the layer resistance *R_s_*, the distance between the electrodes *l*, the cross-section area of the layer A, and the conductivity σ was further calculated by Equation (4)

(4) $$\sigma =\frac{l}{R_{s}\cdot A}$$

It was not possible to conduct the percolation study on the membranes prepared by spin coating due to the small layer thickness. Therefore, wet layer heights of 0.5 and 1 mm were prepared, utilizing the dry casting method, where the dispersion was applied into a frame with the desired layer height. The excess material was then be scraped off and the frame was removed before the drying of the layers. The layers were dried in a vacuum for 24 h. To mitigate the effects of contacting resistance, the contacting areas of each electrode were coated with conductive silver paste before the measurements.

For the percolation study, the additional dispersions with a 5% CNT concentration regarding the dry mass were prepared by both the RS and TRM methods. Both methods were investigated separately using the design of the experiments. The factors that were varied for dispersions with both methods are shown in Table 2.

## 3. Results and Discussion

### 3.1. The Dispersion/Percolation Study

The electrical resistivity of both the MWCNT containing polymer solution (referred to here as the CA system) and the applied layer (similar to MMM) was measured to get a quantitative assessment criterion of dispersion and agglomeration/entangling during the membrane preparation process.

The electrically conductive additives in a non-conductive matrix showed a steeply increasing electrical conductivity after passing the so-called percolation threshold, where the more electrically conductive paths of the additive can be found in the multi-phase system and the shorter the continuous 3D pathways are to bridging the distance between the electrodes, the lower the electrical resistivity is. If the MWCNTs exist as big agglomerates, high additive concentrations are needed to connect them. In contrast, the percolation threshold is much lower for a good dispersion that provides a large number of small agglomerates and even singular MWCNTs with a high aspect ratio. In the middle range, two situations have to be distinguished: a homogenous distribution of MWCNT agglomerates that are not too small, as well as a less homogenous distribution of small agglomerates and (due to the milling effect) shortened MWCNTs that will provide the thresholds in the mentioned middle range [32].

**Figure 3:**
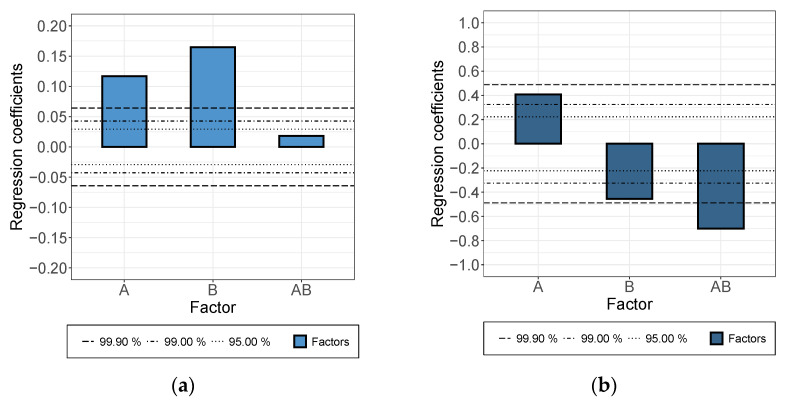
Effects (and confidence intervals) of factors A, B, AB on electrical conductivity of (**a**) the suspension and (**b**) the formed dry layer for rotor-stator dispersing step. A: increasing rotational speed, B: increasing treatment duration, AB: interaction.

The effect of increasing the rotational speed for the rotor-stator dispersing step (parameter “A” in Figure 3) was positive for both the suspension and the applied layer, though the significance of the effect was higher in the case of the suspension (in consideration of confidence intervals). An increased treatment duration (“B”) increased the conductivity in the suspension, but in the applied layer, the opposite effect was detected. Moreover, a strong interaction was monitored. 

**Figure 4:**
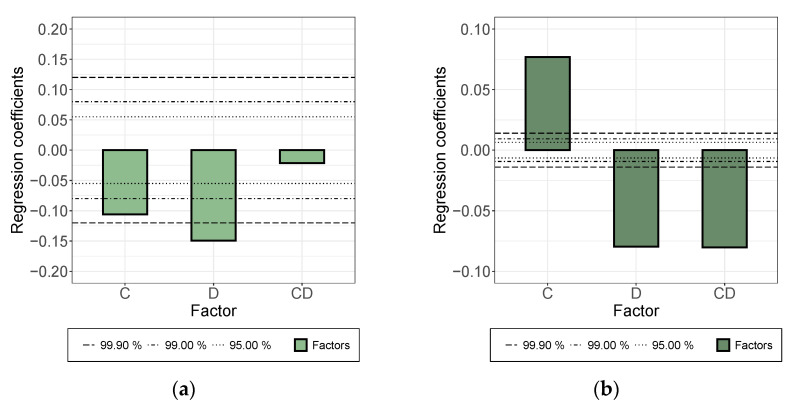
Effects of factors C, D, CD, on electrical conductivity of (**a**) the suspension and (**b**) the formed dry layer, for three-roll-mill dispersing step. C: increasing rotational speed of roller, D: increasing gap width, CD: interaction.

Increasing the rotational speed of the roller for the three-roll-mill dispersing step (“C”, Figure 4) decreased the electrical conductivity in suspension, but increased the conductivity of the applied layer. In the case of the gap width (“D”), the effect was consistent and strongly significant, where narrowing the gap width improved electrical conductivity and widening led to decreasing conductivity. 

In the dispersion/percolation study we examined the influence of the different dispersion methods, RS and TRM. We also investigated the change in the setting parameters.

In some cases, the effects in the suspension and the layers produced from it were uniform, where the increase in speed for RS dispersion and a reduction in the gap width in the TRM dispersion makes sense in every case in terms of increased conductivity and dispersion quality.

In two cases, however, a nonuniform behavior was observed:

1. While a longer treatment time in the rotor-stator increased the conductivity in the dispersion, it decreased in the applied layer. The comminution of the agglomerates (positive) was evidently overcompensated here by a shortening of the MWCNTs and this became effective during the rearrangement during drying. This was also indicated by the significant interaction between parameters A and B.
2. An increase in roller speed in the TRM worsened the conductivity in the suspension, but increased it significantly in the applied layer. The reason for this opposite trend has not yet been clarified. The starting point could be the structural viscosity effects of the suspension in which the increased roller speed reduced the viscosity due to strong shear, so that there was less agglomerate shear. As they dry, the MWCNTs can rearrange themselves and, due to the smaller volume, can result in a higher probability of the connection between MWCNTs.

**Figure 5:**
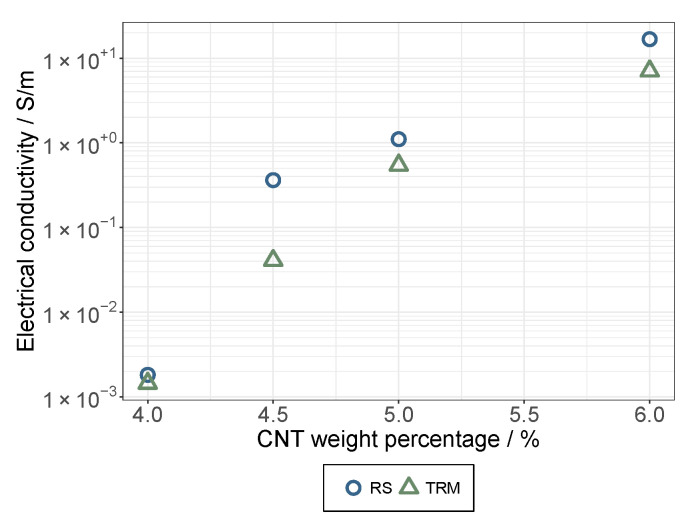
Percolation curves of dried films (CA system), prepared with RS and TRM.

As depicted in Figure 5, the prepared dried CA composite films exhibited an appreciable electrical conductivity. This observation is in good agreement with recent findings reported by Earp et al. [33] regarding the high conductivity of CNT composites below the theoretical percolation values (9–18%). Furthermore, a strong effect when increasing the CNT content in a very narrow window between 4.0 and 6.0 wt.% was observed. Regardless of the dispersion method, the electrical conductivity increased by several orders of magnitude.

For the rotor-stator method, the percolation curves were slightly shifted towards the lower necessary CNT proportions. This is an indication that, with the current parameters, dispersion by means of a rotor-stator system has slight advantages.

### 3.2. Pre Tests on Spin Coating: The Establishment of a Novel Application Method for MMMs

#### 3.2.1. Cellulose Acetate System

We prepared a 10% CA solution and dispersed 2% MWCNT (regarding the dry mass) using the RS at a speed of 6000 min^−1^ for 30 min. For further experiments, the density of the dispersion was measured using a volumetric flask and analytic balance. The density determined at 25 °C was 955.1 kg m^−3^ ± 2.65 kg m^−3^.

For coating on the PP carriers, a short parameter study was conducted regarding the coating speed and the volume of dispersion that was applied to the carrier. The design of the experiments and their later evaluation were both done using the package, rsm, in R [34]. To consider nonlinear correlations, a central composite design was chosen [35]. For the orthogonality and rotatability of the design, a value of α = 1.4141 was chosen, in line with the literature [35] and the rsm package [34]. The parameters and their values are further described in Table 3.

Overall, 48 experiments were conducted for the coating of the PP carriers and each experiment was repeated at least three times. The mass of the wet layer was only affected by the rotational speed, which showed a strong, negative effect with a nonlinear, positive fraction. The volume of dispersion had no detectable effect; thus, there also were no interdependences between speed and volume. This result fits well with the literature, where previous studies concluded that the amount of solution dispersed had no significant impact as long as enough solution was used [17,20].

**Figure 6:**
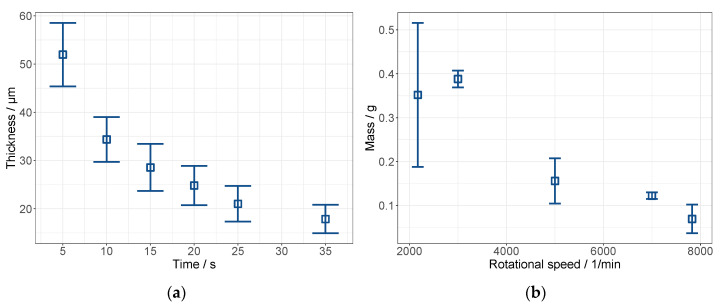
Wet film thickness in dependence of coating time at 4000 rpm coating speed (**a**) and wet mass (**b**) at different coating speeds and coating time of 1 min.

In the first experiments, the time dependence of the layer thickness was determined by interrupting the coating process after certain time increments. The carriers were weighed before and after coating and the grammage and layer thickness were calculated using the carrier’s area and the density of the dispersion. For example, an experiment at 4000 rpm coating speed with 1 mL dispersion on the PP carrier is illustrated in Figure 6a.

To further analyze the effect of coating speed on wet layer mass, experimental results are shown in Figure 6b. As predicted by the parameter study, the amount of dispersion that remained on the carriers decreased with an increasing coating speed. In spin coating, the amount of material that was discharged from the carrier and, thereby, the layer thickness, was determined by the equilibrium between centrifugal force and resistance caused by the viscosity of the fluid [14]. At higher coating speeds, the equilibrium was shifted to thinner layers due to the higher centrifugal force.

From the results in Figure 6b, layer thicknesses were calculated and are shown in Table 4. Additionally, the membranes were weighted after drying to calculate the dry layer weight. A minimum layer thickness of 1.4 ± 0.7 µm was achieved.

It was observed that some of the layers separated from their carriers and some formed small holes after drying. Separation was present mostly with the thicker layers, coated at lower speeds. Thinner layers separated less often; instead, they rolled up together with the carriers. Separation, as well as rolling up, might be explained by the tension induced by drying. In the thicker layers, a stronger force was generated that potentially overcame the adhesion to the carrier’s surface. The detected defects that can be avoided (i.e., separation) by higher rotational speeds, as well as the visible defects (i.e., holes), can be easily rejected in the membrane preparation process. 

After drying, a microscopic study was conducted using optical microscopy. The area and the number of agglomerates were determined with ImageJ Fiji. To analyze and compare results, three parameters were calculated from the measured values: the median agglomerate area, the number of agglomerates per unit of area, and the relative agglomerate area. The relative agglomerate area is defined as the ratio between the sum of all agglomerate areas and the total viewed area. It has to be mentioned, though, that singular CNTs, or very small agglomerates, cannot be captured anymore. Results are shown in Table 5.

Compared to the dispersion quality achieved by the RS, samples prepared using the TRM seemed to have slightly bigger, but fewer, agglomerates. It has to be considered that the median agglomerate area was skewed towards bigger values due to the limited resolution of the microscope used. A dispersion of a higher quality has less detectable agglomerates. For this reason, the relative agglomerate area was introduced as an added value to assess the dispersion quality, regarding the size and the number of agglomerates. This new value also implied that a superior dispersion quality might be achieved using the TRM over the RS. However, it was difficult to replicate the results from the experiments applying the TRM, as indicated by the errors stated in Table 5. This could be explained by the overall higher complexity of the TRM. A more extensive investigation of the parameters influencing the dispersion process with the TRM is necessary to achieve higher levels of repeatability.

Ahmad et al. [12] found similar sized agglomerates in membranes prepared from the CA polymer with nonmodified MWCNTs as well. However, the comparability to the results presented here is limited, because different concentrations of MWCNTs, solvents, and preparation processes were applied in the literature.

#### 3.2.2. Polyimide System

The coating of the dispersion made from the PI solution was also analyzed in a parameter study. The effects of the coating speed, polymer concentration, and the coating time were examined using a central composite design. Regarding the results from the aforementioned study on the coating of CA-based dispersion, the applied volume was fixed at 2 mL. The dispersions had a MWCNT concentration of 2 wt.% regarding the dry mass and were dispersed using the RS at 6000 rpm for 30 min. Parameters and their values are shown in Table 6.

**Figure 7:**
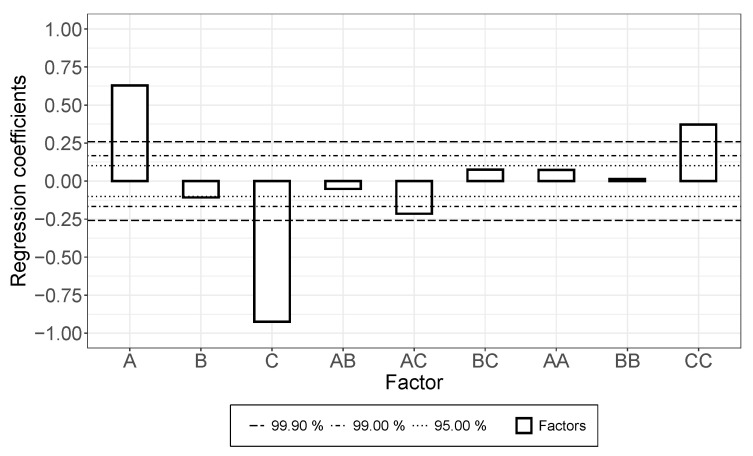
Effects of polymer concentration (A), coating time (B), coating speed (C), interdependences, and nonlinear effects on dry layer thickness in coating of PI-based dispersions.

The results of this study are illustrated in Figure 7. As seen in the study regarding the coating of the CA dispersion, the coating speed had a strong negative effect with a nonlinear, but positive, fraction, which means a disproportionate rise. The coating time only had a weak, non-significant, negative effect. The concentration of the polymer solution had a strong positive effect, which was caused by the increase in viscosity (Section 3.3) that comes with higher concentrations. The results also showed an interdependence between polymer concentrations and the coating speed. This indicates a decrease in layer height if the concentration and the coating speed are increased at the same rate.

**Figure 8:**
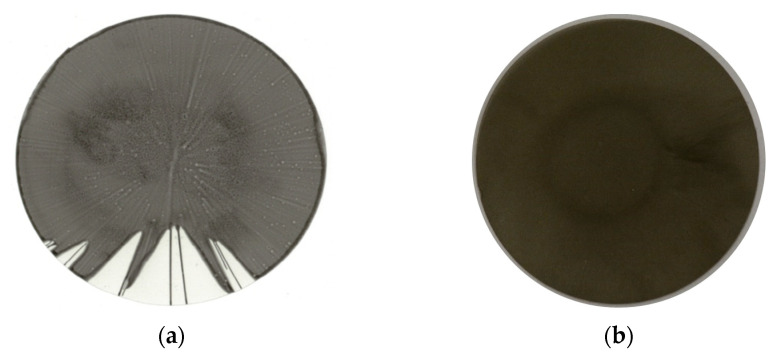
PI-based layer from solution with 12.7 wt.% polymer concentration, prepared at an insufficient coating speed of 1000 rpm for 150 s (**a**) compared to a membrane with improved surface coverage (**b**).

To determine the set values for the parameters used in the coating studies, preliminary tests were conducted. For both systems, the most critical conditions were found at low coating speeds and small applied amounts. Figure 8 shows an example of a carrier, which was incompletely coated due to a low rotational speed. Additionally, strands can be observed on the coated areas, which indicates an insufficient amount of dispersion applied. 

It was found that the sufficient coating speed to achieve the complete coverage of the carrier’s surface would be at least 2000 rpm. At lower speeds, the dispersion would flow off the carrier in channels (Figure 8a) and the material between these channels would stay uncovered, making it impossible to prepare a gastight layer. The maximum coating speed was found at about 8000 rpm. It was possible to produce completely covered carriers at higher speeds, but the layers would become so thin that they tended to rip and form cracks during drying.

The amount of dispersions applied to the carriers with a diameter of 10 cm should not be smaller than 1 mL, and for the preparation of layers on PES carriers, 2 mL were used so as to not risk any damages. At lower applied amounts, the increased formation of small holes and strands on the surfaces of the layers was observed. The amount applied to the carrier should not be too big either, as the excess dispersion is discharged and found on the walls of the spin coater. 

### 3.3. Rheological Analysis

**Figure 9:**
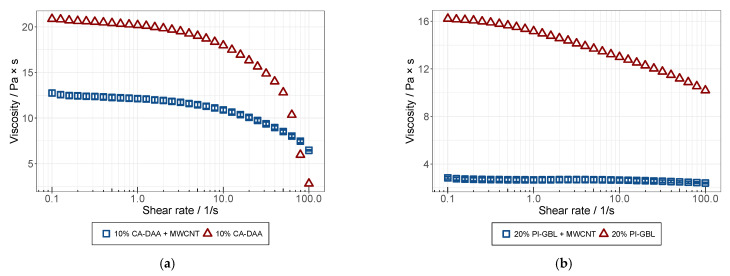
Viscosity curves of solutions and dispersions from CA (**a**) and PI (**b**) measured at 25 °C with plate-plate configuration rheometer.

Viscosity curves for the solution and dispersions prepared from both polymers were analyzed with the rheometer, Kinexus Pro (Malvern, Kassel, Germany), in a plate-plate configuration at 25 °C. In this experiment, the shear stress was measured at different shear rates ranging from 0.1–100 s^−1^. The dynamic viscosity was then calculated from the shear stress values. In comparison, the viscosity curves of the CA- and PI-based dispersions are illustrated in Figure 9 together with the viscosity curves of the polymer solutions they were prepared from. Surprisingly, for both polymers, the dispersions showed an overall lower viscosity than the polymer solutions. This could be caused by the solution blending method, where an additional solvent was introduced together with the MWCNTs, further diluting the solutions. An increased amount of the additional solvent was added to the PI solution because the dispersions were prepared with a mass fraction of 2% MWCNT, referring to the mass of the polymer in the solution. Therefore, greater amounts of the MWCNT predispersion was added, due to the higher concentration of the polymer in the PI solution used for the experiments. Both solutions exhibited shear-thinning behaviors, which weakened (CA) or disappeared (PI) after the further processing of the dispersions, as it seemed to exceed the dilution effect of the polymer. Both solvents reduced the structural viscose effect of the polymer solution. The influence of the viscosity due to the MWCNT, because of its low concentration (0.18 wt.%), should be low. Further polymer concentrations were analyzed with the same effect, but the coating of membranes could only be successfully achieved by higher viscosities. Therefore, we focused on the concentrations shown in Figure 9. The shear-thinning behavior found is typical for polymeric solutions and is caused by the alignment of polymer chains by shear flow [36,37].

### 3.4. Membrane Preparation

The preparation of actually permeable membranes was done in a similar way to the spin coating experiments, but instead of PP films, porous carriers were used. For the CA-based dispersions, a PES membrane, Virosart (Sartorius), was chosen due to its high permeability for the test gases and the small pore size. 

**Figure 10:**
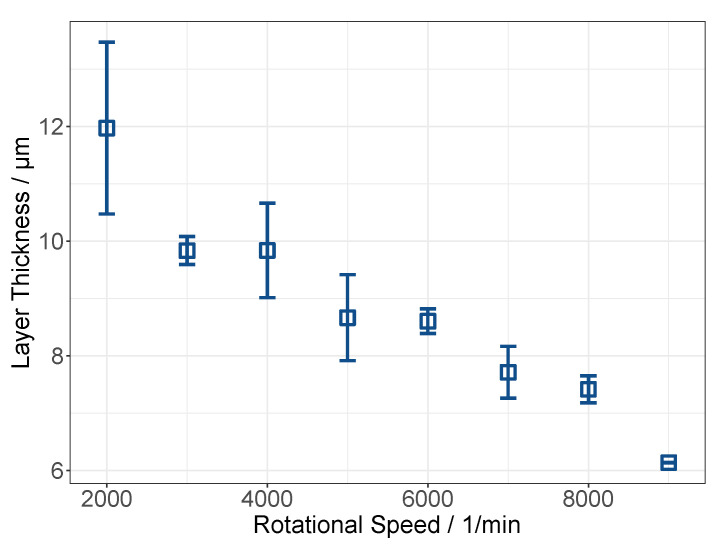
Layer height of CA dispersion on PES carriers at different coating speeds, with coating time of 1 min.

As a first step, the coating behaviors of the CA-based dispersions on the PES carriers were analyzed, similar to the procedure from Section 3.1, by measuring the layer thickness at various coating speeds. For better comparability, the dispersion for this experiment was prepared at the same parameters used for the dispersion coated on the PP carriers, with the RS at 6000 rpm for 30 min. The results are in accordance with the pretests in Section 3.2, illustrated in Figure 10.

On the PES carriers, thicker layers were generated by spin-coating. This could be explained by differences in the surface adhesion between the dispersion and carrier materials. To check this hypothesis, the contact angles of the dispersion on both carriers were measured with a goniometer, G-1 (Krüss, Hamburg, Germany). The resulting contact angles were 64.3 ± 4.5° for the dispersion on the PES membranes and 40.3 ± 2.3° on the PP carrier. These findings contradict the effects of surface attraction on the observed differences in layer thicknesses. 

It was also observed that the PES carrier membranes were fully wetted after spin coating. An additional investigation regarding the wetting behaviors showed that the PES membrane absorbed DAA. However, in a microscopic analysis of the finished membranes backsides, no signs of dispersions that could have penetrated the membranes were found. This leads to the assumption that the solvent is separated from the dispersion during spin coating, causing an increase in viscosity and, therefore, the formation of thicker layers.

The coated membranes were dried at room temperature for 24 h on a stainless steel plate. To prevent them from rolling up, they were clamped down using holding rings that only had contact with the outer edges of the membranes. Using this method, the damage to the main parts of the membrane were avoided. After drying, the membranes were stored between PP films.

**Figure 11:**
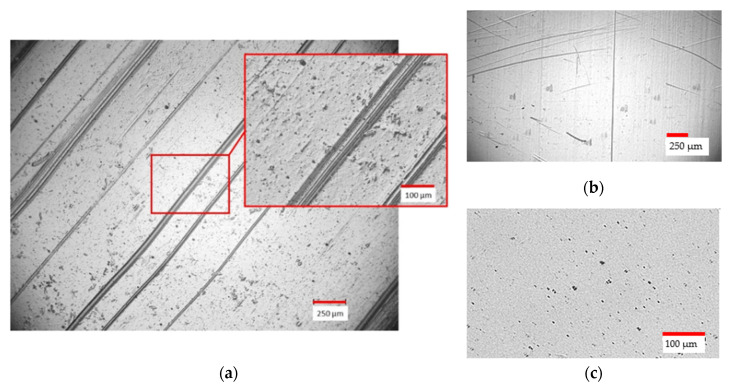
Irregularities found on the CA membrane surfaces after coating and drying. A pattern of defects with a close-up (**a**). In hindsight, the defects were also found on the non-coated PES carrier (**b**), but, as a consequence, the CA membrane, without visible defects, could be produced (**c**).

On some of the membrane surfaces, linear defects became visible after drying. A typical pattern of the observed scratch marks is shown in Figure 11a, as well as a close-up view of a single defect. The defects were also found on the PES carrier membranes, after a closer inspection. All further membrane preparation carriers were checked before coating to avoid the damaged ones. However, the permeability tests showed that the defects did not affect the functionality. This leads to the assumption that the dispersion fills the defects on the carrier membrane. 

The PI dispersion could not be coated onto the PES carrier membranes due to the poor chemical resistance of the membranes against GBL, which was used to dissolve the polymer. Therefore, a material with better resistance was found and the application of two types of porous membranes were made from PP, with pore sizes of 0.6 and 0.2 µm, and were tested as alternative carriers. The first coating experiments showed that the dispersion soaks into the membranes and even leaks through the 0.6 µm membranes. Due to the leaking, the dispersion would not form a uniform layer like it would on the PES carriers. An additional method was tested where the membranes were coated a second time after drying. Here, the assumption was made that the dispersion would saturate the membrane and block the pores, allowing the second dose of dispersion to form the desired, uniform layer. By this method, the seepage through the membranes was prevented. However, the formed layer still exhibited some irregularities and defects. On some of the wet layers, puddles of dispersion formed, which indicated the poor comparability of the dispersion and the carrier. Contact angles could not be measured on the PP membranes because the drops of dispersion were absorbed by the membrane too quickly.

To improve the surface properties and the wettability of the membranes, they were exposed to a plasma treatment using a low-pressure microwave plasma with argon/oxygen mixtures. Plasma-treated membranes were coated with the same methodsas the untreated membranes. For the single-coated membranes, an increase in leakage was observed, and the membranes with 0.2 µm pore size leaked as much as the 0.6 µm membranes. After drying and coating again, uniform layers were formed on the membranes. Before the further analysis of the coating process, a permeation analysis of the membranes from the first tests was conducted. Additional membranes were prepared with three to four coating steps. 

### 3.5. Plasma Treatment

Plasma processes were especially suitable for modifying polymer surfaces, providing them with functional groups and, thus, changing the surface properties [38]. Spectroscopic measurements that were conducted after processing in an Ar/O_2_ microwave-induced plasma revealed the presence of several argon and oxygen species [39], such as radicals, atoms, and ions. They interacted with the polymer surfaces, inducing chemical reactions and forming polar hydrophilic O-containing functional groups in the uppermost layer of the polymers [40,41]. 

**Figure 12:**
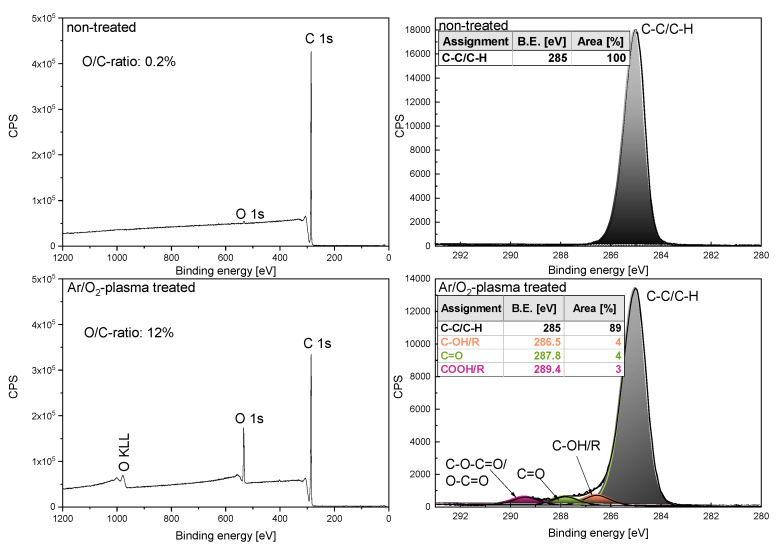
XPS analysis of non-treated and Ar/O_2_-plasma treated polypropylene samples (**top**: non-treated control, **bottom**: Ar/O_2_-plasma treated). Survey scans (**left**) confirmed the presence of carbon and oxygen on the surfaces. Highly resolved and measured C 1s spectra (**right**) demonstrated the formation of polar functional groups after plasma treatment.

In Figure 12, the results of the XPS analysis of the non-treated polymer before and after Ar/O_2_-plasma treatments are shown. The non-treated PP surface contained mainly carbon (approximately 99.8%) and only traces of oxygen (0.2%). After the treatment in Ar/O_2_-plasma in the survey scan, an increased amount of oxygen (approximately 11%) and a reduced amount of carbon (approximately 89%) with an O/C element ratio of 12% was observable. 

The highly resolved measured C 1s spectra of the non-treated PP material was fitted using only the aliphatic bonded carbon component. After the Ar/O_2_-plasma treatment, the peak shape changed due to the insertion of O-containing functional groups, such as hydroxyl (approximately 4%), carbonyl (approximately 4%), and carboxyl groups (approximately 3%) as can be seen in Figure 12 (bottom, right). Thus, the surface was functionalized with hydrophilic groups, which caused an improved wettability. 

However, the hydrophilization was, typically, not stable. Indeed, the plasma functionalized surfaces were prone to aging effects due to a partial or complete hydrophobic recovery. The reorientation of the surface layer and the migration of the polymer chains from the bulk area to the surface, or vice versa, were identified as the main reasons for this recovery process on dry storage [42].

The carriers were coated 24 and 48 h after the plasma treatment by the same method used for the untreated carriers. In the meantime, they were stored at room temperature. The carriers coated after 24 h all exhibited leakage, which was only observed on the 0.6 µm carriers before, and it was not possible to create uniform layers. For the carriers that were coated 48 h after treatment, on the other hand, an improved coating behavior was observed on some of the carriers. However, no sufficient reproducibility could be achieved with this process.

**Figure 13:**
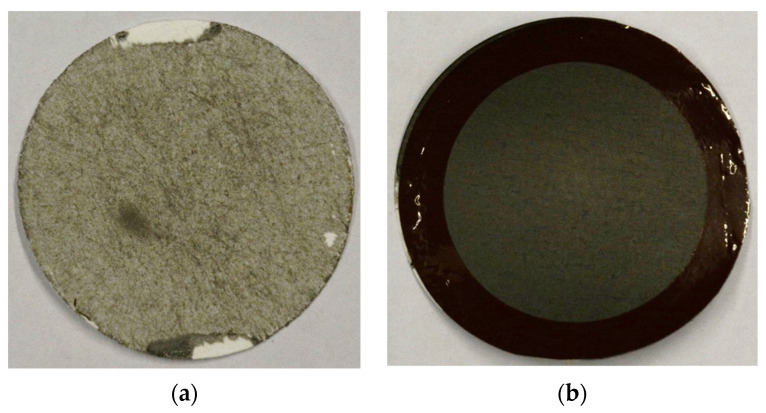
Comparison between membranes coated on untreated (**a**) and treated (**b**) carriers.

Additional experiments were conducted with a longer delay between the plasma treatment and the coating of the carriers with multiple coatings. With the longer waiting time, no additional effects on the coating behaviors were found, even after 21 days. By applying multiple layers, a visible improvement of the membrane surface was achieved. However, the leakage through the carriers could not reliably be prevented. The gas separation performance of the membranes with a good surface quality was analyzed. The comparison of the surface structure membranes prepared from untreated and treated carriers are illustrated in Figure 13.

The presence of carbonyl, carboxy, and hydroxyl functional groups on the surface after the plasma treatment led to a higher hydrophilic surface. In combination with the porous structure of the carrier, the dispersion was completely soaked in the porous structure. For the PES-CA membranes, the higher viscosity prevented this soaking effect observed by PI. For good coating behaviors, the spin coating process required a smooth surface, but due to the soaking of PI, uniform distribution could not be obtained. This effect led to the observations described above. However, multiple coatings results in a dense active membrane layer which prevents the permeation of gases. 

### 3.6. Permeation Analysis

The gas permeation properties of the membranes prepared by spin coating were conducted using the testing setup illustrated in Figure 2. As a first step, the general gas tightness of the membranes were tested with N_2_ to analyze the effects of the layer thickness on defects. For comparison, the permeability of CO_2_ was also measured. Its high permeability through both polymers [1,6] and its selectivity was calculated. The gas separation performances of the mixed matrix membranes were compared to pristine polymer membranes that were prepared using the same process but without MWCNTs. The permeability was calculated from the incline of the permeate pressure over time by Equation (5) [43], where *l* is the layer thickness in cm, *A* is the membrane area in cm^2^, *V* is the volume of the vacuum cavity in cm^3^, *p_F_* is the feed pressure in bar, and *T* is the temperature in Kelvin. The unit of the calculated permeability *P_i_* is Barrer.

(5) $$P_{i}=3.59\cdot 10^{10}\cdot \frac{l\cdot V}{p_{F}\cdot A\cdot T}\cdot \frac{dp}{dt}$$

**Figure 14:**
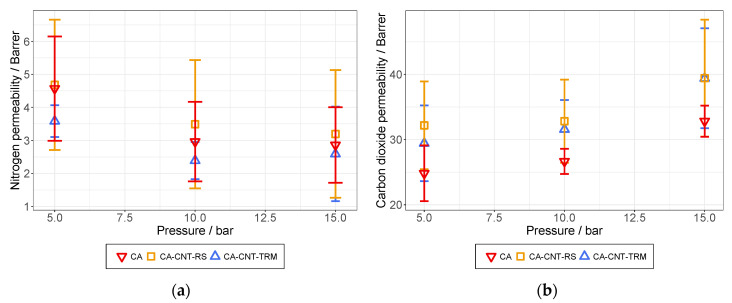
Gas permeation at feed pressures of 5, 10, and 15 bar measured with N_2_ (**a**) and CO_2_ (**b**).

The first tests with the CA-based membranes showed that all membranes had a low permeability to N_2_. No effects of the layer thickness were found and even the membranes with visible surface defects showed no reduced gas tightness. Permeabilities measured at feed pressures of 5, 10, and 15 bar are illustrated in Figure 14a. It can be seen that the permeability declines with increasing pressures. For comparison, the measured values of CO_2_ permeation are shown in Figure 14b. Unlike the N_2_ permeability, the CO_2_ permeability rose with increasing pressure. This was also observed by Moghadassi et al. [44] analyzing similar types of membranes. It can be explained by the pressure dependency of CO_2_ solubility in CA [45].

**Figure 15:**
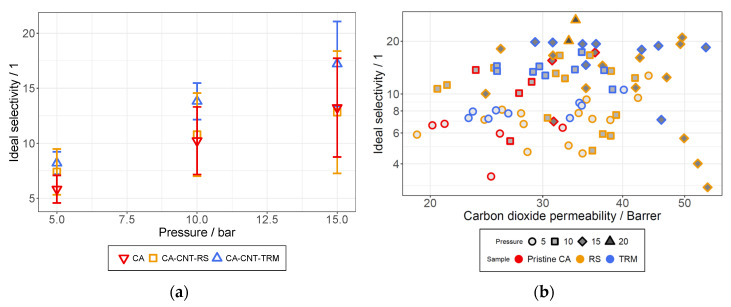
Selectivity of CA membranes at 5, 10, and 15 bar (**a**) and the selectivity against the permeability in dependence of the pressure 5, 10, 15, and 20 bar (**b**).

Additional experiments were conducted at a feed pressure over 15 bar to identify the maximum pressure the membrane could withstand. It was found that half of the membranes failed at 20 bar, mostly by ripping in the area near the outer edge of the testing module. For the membranes that tolerated the higher pressure, the raised selectivities were measured (Figure 15b).

For the membranes prepared from the RS dispersions, a significant deviation was found. This might be explained by aging effects, because some of the said membranes could not be analyzed immediately after their preparation. The membranes were stored for one week between PP films, protected from direct sunlight and moisture. After one week, they exhibited a significantly lower permeation for both test gases. A possible explanation for this behavior might be the unknown effects that were caused by the dispersed MWCNTs on the surrounding polymer matrix. In future experiments, the aging behavior of mixed matrix membranes and pristine polymer membranes will be compared to verify this hypothesis.

From the results of the permeation experiments, selectivities were calculated via Equation (6), where *P_i_* is the permeability of the gas that passes through the membrane (CO_2_) and *P_j_* is the permeability of the gas that is primarily retained (N_2_). Calculated selectivities at different feed pressures are illustrated in Figure 15.

(6) $$\alpha_{ij}=\frac{P_{i}}{P_{j}}$$

From the permeability curves in Figure 14, it can already be interpreted that the selectivity rises with increasing pressure due to the opposite curve of both permeabilities. What also stands out is that the membranes prepared from the TRM dispersions exhibited the highest selectivities while they were astride the membranes from the RS dispersions regarding the CO_2_ permeability. The main cause for the overall good performance of these membranes seems to be the high retention of N_2_ that is indicated in Figure 14a. From Figure 14b, it also became apparent that the CO_2_ permeability could overall be improved by the addition of MWCNTs into the CA membrane. 

**Figure 16:**
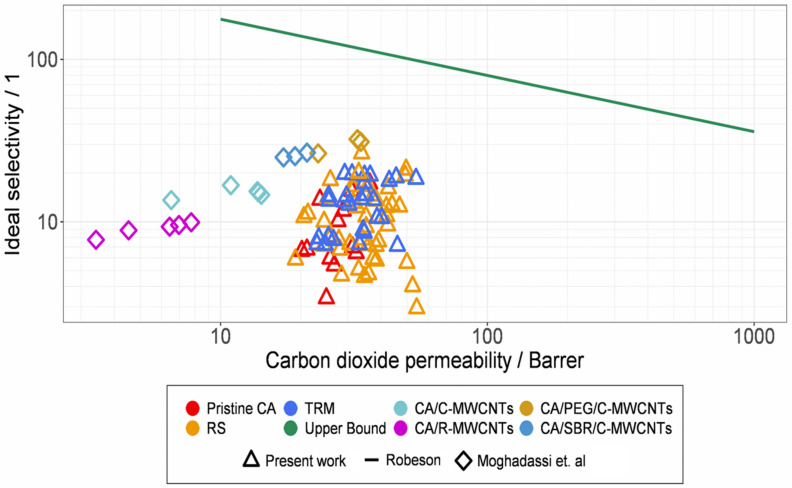
CO_2_ permeability and corresponding CO_2_/N_2_ selectivity in comparison to the Robeson upper bound [6,44].

For an easier comparison, all results from the permeation analysis of the CA-based membranes are shown in Figure 15b. Additionally, the current Robeson upper bond and comparable literature for CO_2_/N_2_ are illustrated in Figure 16. The selectivity to permeability ratios observed for the prepared membranes could not reach the upper bond. However, multiple opportunities for its optimization could still be investigated, e.g., different concentrations of CNTs or combinations of both dispersion methods. In [14] a possible positive effect of ultrasonic treatment was mentioned, which was not investigated in this study.

The permeations of the PI-based membranes were analyzed in a similar way to the CA membranes. However, the evaluation of the results was not possible, because the prepared membranes were either completely impermeable or did not hold back the gases at all. No differences between the permeability of the gases could be detected; therefore, the selectivities could not be calculated. PI-based MMMs still suffered from a narrow window of the wetting behavior of the carrier, e.g., affected by the use of plasma, which is discussed in the following paragraph. 

### 3.7. Comparisons and Classifications with Former Results

In a previous study [13], we compared the dispersion quality, permeability, and selectivity of membranes redispersed with the RS and the ultrasonic sonotrode (USS). It showed that the RS is a suitable method, compared to the USS. The permeability of CO_2_ was increased from the previous work, where we achieved an improvement in permeabilities of 3- to 8-times higher. This improvement resulted in a better coating method due to the use of spin coating and, therefore, a thinner active layer. In addition, the spin coater enabled the coating on a support layer and increased the operating pressures.

## 4. Conclusions and Outlook

Our study shows that the integration of the MWCNTs increases the permeability of CO_2_. We further identified the influence of the dispersing method of gas separation. Unfortunately, we couldn’t surpass the Robeson upper bound, as Figure 16 depicts. In comparison to other methods, our path requires few resources. We increased the permeability and selectivity of CA by redispersing MWCNT in our polymeric solution. This shows that the TRM increases the selectivity, whereas the RS increases the permeability. However, the shortening of the MWCNT has a negative effect on gas separation. Based on this insight, we want to combine the RS and the TRM to use the efforts of both.

Furthermore, we want to optimize the coating of the PI system to analyze the permeability and selectivity of this membrane type. Therefore, we want to examine the interaction of the plasma-treated layer and our cast solution to identify the critical parameters for the preparation of a suitable membrane.

Due to the simplicity of our membrane preparation method, we can easily investigate several other dispersion processing options to increase the distribution of the MWCNTs and, thus, the permeability and selectivity. However, the spin coating method is not suitable for the production of industrial-scale membrane sheets, and the process presented in this work is intended to allow for quick screening experiments in laboratory-scale applications. Therefore, the estimation of the final cost per area unit of the membranes is difficult and the process should first be compared to an industrial membrane preparation process.

Long-term stability, especially under the influence of CO_2_, could also be investigated in future experiments. The experimental setup constructed for this work could be modified to allow this kind of experiment, with little expenditure.

Additionally, we hope to optimize the CA system, and, thus, increase the pressure up to 20 bar for the permeation analysis. For this, the optimization of the coating process is required. The plan to improve the CA system is by adding multiple active layers. If necessary, combining different MWCNT concentrations in a single layer to optimize the gas transport, and also the mechanical properties, could also be performed.

Finally, we want to determine the selectivity for binary gas mixtures to confirm the selectivities of this work. Moreover, we plan to investigate the permeation of water to compare with the results from previous work [13], as well as investigating additional gases, such as O_2_ and He, to further characterize our membranes.

## Figures and Tables

**Table 1:** Concentrations of polymer, solvent, and CNTs in dispersions prepared from polymer solutions and 1.5 wt.% CNT predispersion for membranes with a CNT concentration of 2 wt.% after drying.

Polymer in Solution	Polymer in Dispersion	Solvent in Dispersion	CNT in Dispersion
PI 20%	15.79%	83.89%	0.32%
CA 10%	8.82%	91.0%	0.18%

All percentages in wt.%.

**Table 2:** Factors used in dispersion preparation for percolation study.

Three Roll Mill		Rotor-Stator	
Rotational Speed min^−1^	Gap Width µm	Rotational Speed min^−1^	Duration of Treatment Min
100	5	2000	10
300	15	6000	30

**Table 3:** Parameters for parametric study for spin coating on PP carriers.

Norm. Value	Rotational Speed min^−1^	Applied Volume mL
−α *	2172	1
−1	3000	1.5
0	5000	2
+1	7000	2.5
+α *	7828	3

* α = 1.4141.

**Table 4:** Grammage and thickness of dry layers.

Rotational Speed min^−1^	Wet Layer Thickness µm	Dry Layer Thickness µm
2172	46.9 ± 21.9	3.89 ± 2.14
3000	51.8 ± 2.55	4.69 ± 0.36
5000	20.8 ± 6.86	2.23 ± 0.97
7000	16.3 ± 0.98	1.54 ± 0.13
7828	9.27 ± 4.34	1.4 ± 0.7

**Table 5:** Results of microscopic analysis of CA samples on PP carriers.

Dispersion Method	Rotor Stator	Three-Roll-Mill
Median agglomerate size	1.38 ± 0.075 µm	1.58 ± 0.51 µm
Number of agglomerates	92.3 ± 19.6 × 10^−3^ mm^−2^	29 ± 10 × 10^−3^ mm^−2^
Relative agglomerate area	14.0 ± 3.8%	6.17 ± 4.57%

**Table 6:** Parameters for study on coating of PI-based dispersions.

Norm. Value	Rotational Speed min^−1^	PI-Concentration wt.%	Coating Time s
−α *	1734	12.37	21
−1	3000	13	30
0	5000	14	45
+1	7000	15	60
+α *	8266	15.63	69

* α = 1.63.

## Data Availability

Not applicable.

## References

[1] George G., Bhoria N., AlHallaq S., Abdala A., Mittal V. (2016). Polymer membranes for acid gas removal from natural gas. Sep. Purif. Technol..

[2] Nakao S.-I., Yogo K., Goto K., Kai T., Yamada H. (2019). Advanced CO_2_ Capture Technologies.

[3] Rezakazemi M., Ebadi Amooghin A., Montazer-Rahmati M.M., Ismail A.F., Matsuura T. (2014). State-of-the-art membrane based CO_2_ separation using mixed matrix membranes (MMMs): An overview on current status and future directions. Prog. Polym. Sci..

[4] Wang Y., Wang X., Guan J., Yang L., Ren Y., Nasir N., Wu H., Chen Z., Jiang Z. (2019). 110th Anniversary: Mixed Matrix Membranes with Fillers of Intrinsic Nanopores for Gas Separation. Ind. Eng. Chem. Res..

[5] Chung T.-S., Jiang L.Y., Li Y., Kulprathipanja S. (2007). Mixed matrix membranes (MMMs) comprising organic polymers with dispersed inorganic fillers for gas separation. Prog. Polym. Sci..

[6] Robeson L.M. (2008). The upper bound revisited. J. Membr. Sci..

[7] Vu D.Q., Koros W.J., Miller S.J. (2003). Mixed matrix membranes using carbon molecular sieves. J. Membr. Sci..

[8] Moore T.T., Mahajan R., Vu D.Q., Koros W.J. (2004). Hybrid membrane materials comprising organic polymers with rigid dispersed phases. AIChE J..

[9] Asif K., Lock S.S.M., Taqvi S.A.A., Jusoh N., Yiin C.L., Chin B.L.F., Loy A.C.M. (2021). A Molecular Simulation Study of Silica/Polysulfone Mixed Matrix Membrane for Mixed Gas Separation. Polymers.

[10] Wong K.K., Jawad Z.A., Chin B.L.F. (2021). A polyethylene glycol (PEG)—Polyethersulfone (PES)/multi-walled carbon nanotubes (MWCNTs) polymer blend mixed matrix membrane for CO_2_/N_2_ separation. J. Polym. Res..

[11] Juber F.A.H., Jawad Z.A., Teoh G.H., Ahmad A.L., Chin B.L.F. (2021). Development of novel blend poly (Ethylene Glycol)/Poly(Ethersulfone) polymeric membrane using N-Methyl-2-Pyrollidone and dimethylformamide solvents for facilitating CO2/N2 gas separation. Mater. Today.

[12] Ahmad A.L., Jawad Z.A., Low S.C., Zein S.H.S. (2014). A cellulose acetate/multi-walled carbon nanotube mixed matrix membrane for CO2/N2 separation. J. Membr. Sci..

[13] Esser T., Wolf T., Schubert T., Benra J., Forero S., Maistros G., Barbe S., Theodorakopoulos G.V., Karousos D.S., Sapalidis A.A. (2021). CO_2_/CH_4_ and He/N_2_ Separation Properties and Water Permeability Valuation of Mixed Matrix MWCNTs-Based Cellulose Acetate Flat Sheet Membranes: A Study of the Optimization of the Filler Material Dispersion Method. Nanomaterials.

[14] Yonkoski R.K., Soane D.S. (1992). Model for spin coating in microelectronic applications. J. Appl. Phys..

[15] Meyerhofer D. (1978). Characteristics of resist films produced by spinning. J. Appl. Phys..

[16] Emslie A.G., Bonner F.T., Peck L.G. (1958). Flow of a Viscous Liquid on a Rotating Disk. J. Appl. Phys..

[17] Schubert D.W., Dunkel T. (2003). Spin coating from a molecular point of view: Its concentration regimes, influence of molar mass and distribution. Mater. Res. Innov..

[18] Clausi M., Santonicola M.G., Laurenzi S. (2016). Fabrication of carbon-based nanocomposite films by spin-coating process: An experimental and modeling study of the film thickness. Compos. Part A.

[19] Lee U.G., Kim W.-B., Han D.H., Chung H.S. (2019). A Modified Equation for Thickness of the Film Fabricated by Spin Coating. Symmetry.

[20] Burmann P., Zornoza B., Téllez C., Coronas J. (2014). Mixed matrix membranes comprising MOFs and porous silicate fillers prepared via spin coating for gas separation. Chem. Eng. Sci..

[21] (2010). Handbook of Industrial Mixing: Science and Practice.

[22] Håkansson A. (2018). Rotor-Stator Mixers: From Batch to Continuous Mode of Operation—A Review. Processes.

[23] Thostenson E.T., Chou T.-W. (2006). Processing-structure-multi-functional property relationship in carbon nanotube/epoxy composites. Carbon.

[24] Gojny F.H., Wichmann M.H.G., Köpke U., Fiedler B., Schulte K. (2004). Carbon nanotube-reinforced epoxy-composites: Enhanced stiffness and fracture toughness at low nanotube content. Compos. Sci. Technol..

[25] Li Y., Zhang H., Bilotti E., Peijs T. (2016). Optimization of Three-Roll Mill Parameters for In-Situ Exfoliation of Graphene. MRS Adv..

[26] Schröder K., Meyer-Plath A., Keller D., Besch W., Babucke G., Ohl A. (2001). Plasma-Induced Surface Functionalization of Polymeric Biomaterials in Ammonia Plasma. Contrib. Plasma. Phys..

[27] Beamson G., Briggs D. (1992). High Resolution XPS of Organic Polymers: The Scienta ESCA300 Database.

[28] Schindelin J., Arganda-Carreras I., Frise E., Kaynig V., Longair M., Pietzsch T., Preibisch S., Rueden C., Saalfeld S., Schmid B. (2012). Fiji: An open-source platform for biological-image analysis. Nat. Methods.

[29] Lomax M. (1980). Permeation of gases and vapours through polymer films and thin sheet—Part I. Polym. Test..

[30] Naftaly M., Das S., Gallop J., Pan K., Alkhalil F., Kariyapperuma D., Constant S., Ramsdale C., Hao L. (2021). Sheet Resistance Measurements of Conductive Thin Films: A Comparison of Techniques. Electronics.

[31] Miccoli I., Edler F., Pfnür H., Tegenkamp C. (2015). The 100th anniversary of the four-point probe technique: The role of probe geometries in isotropic and anisotropic systems. J. Phys. Condens. Matter.

[32] Buschhorn S.T., Wichmann M.H.G., Sumfleth J., Schulte K., Pegel S., Kasaliwal G.R., Villmow T., Krause B., Göldel A., Pötschke P. (2011). Charakterisierung der Dispersionsgüte von Carbon Nanotubes in Polymer-Nanokompositen. Chem. Ing. Tech..

[33] Earp B., Simpson J., Phillips J., Grbovic D., Vidmar S., McCarthy J., Luhrs C.C. (2019). Electrically Conductive CNT Composites at Loadings below Theoretical Percolation Values. Nanomaterials.

[34] Lenth R.V. (2009). Response-Surface Methods in R, Using rsm. J. Stat. Soft..

[35] Kleppmann W. (2020). Versuchsplanung: Produkte und Prozesse Optimieren, 10, Überarbeitete Auflage.

[36] Schröder T. (2018). Rheologie der Kunststoffe.

[37] Dunstan D.E. (2019). The viscosity-radius relationship for concentrated polymer solutions. Sci. Rep..

[38] Ohl A., Schröder K., Hippler R., Kersten H., Schmidt M., Schoenbach K.H. (2008). Plasma assisted surface modification of biointerfaces. Low Temperature Plasma Physics: Fundamental Aspects and Applications.

[39] Baeva M., Stankov M., Trautvetter T., Methling R., Hempel F., Loffhagen D., Foest R. (2021). The effect of oxygen admixture on the properties of microwave generated plasma in Ar–O_2_: A modelling study. J. Phys. D.

[40] Hempel F., Steffen H., Busse B., Finke B., Barbara J., Quade A., Rebl H., Bergemann C., Weltmann K.-D., Schroder K., Fazel R. (2011). On the Application of Gas Discharge Plasmas for the Immobilization of Bioactive Molecules for Biomedical and Bioengineering Applications. Biomedical Engineering—Frontiers and Challenges.

[41] Mokbul H.M., Dirk H. (2011). Substrate independent dyeing of synthetic textiles treated with low-pressure plasmas. Text. Dyeing. Croat..

[42] Jokinen V., Suvanto P., Franssila S. (2012). Oxygen and nitrogen plasma hydrophilization and hydrophobic recovery of polymers. Biomicrofluidics.

[43] Ebadi Amooghin A., Omidkhah M., Kargari A. (2015). Enhanced CO_2_ transport properties of membranes by embedding nano-porous zeolite particles into Matrimid^®^5218 matrix: Supplementary information. RSC Adv..

[44] Moghadassi A.R., Rajabi Z., Hosseini S.M., Mohammadi M. (2014). Fabrication and modification of cellulose acetate based mixed matrix membrane: Gas separation and physical properties. J. Ind. Eng. Chem..

[45] Stern S.A., de Meringo A.H. (1978). Solubility of carbon dioxide in cellulose acetate at elevated pressures. J. Polym. Sci. Polym. Phys. Ed..

